# P10 Risk management of clinical false positives with multiplex PCR blood culture panels: a lesson in diagnostic stewardship for microbiology trainees

**DOI:** 10.1093/jacamr/dlae136.014

**Published:** 2024-08-23

**Authors:** S Gregg, C Mulrooney, M Leonard, M Flanagan, D Nashev, M Cormican

**Affiliations:** Galway University Hospital, Galway, Ireland; Galway University Hospital, Galway, Ireland; Galway University Hospital, Galway, Ireland; Portunicula University Hospital, Ballinasloe, Ireland; Galway University Hospital, Galway, Ireland; Portunicula University Hospital, Ballinasloe, Ireland; Galway University Hospital, Galway, Ireland; University of Galway, Galway, Ireland

## Abstract

**Background:**

The processing of blood cultures is technically difficult and time consuming. Nucleic acid amplification tests (NAATs) can supplement this process, with some commercial systems offering NAAT for multiple pathogens simultaneously. These platforms also present distinct challenges, including managing false positive identifications. The use of broad multiplex NAAT in cases of low pre-test probability carries an inherent risk of low positive predictive value. We describe a series of detections of *Candida tropicalis* targets from positive blood cultures where *C. tropicalis* was not present and subsequent management.

**Incident:**

Biofire Filmarray BCID2 was introduced in May 2022 in a General Hospital to aid laboratory workflow. The BCID2 panel is used 5–10 times weekly on positive blood cultures. BC specimens are then sent to an associated hospital for sub-culture, identification and antimicrobial susceptibility testing. In August 2023 a blood culture with Gram-positive cocci on Gram stain was positive for *C. tropicalis and Staphylococcus* spp. targets on BFBCID2. The consultant microbiologist did not advise antifungal treatment based on Gram stain and assessment of patient clinical features. Only *Staphylococcus* spp. was identified on culture. bioMérieux UK Ltd subsequently released a Field Safety Notice in August 2023 highlighting the risk of false positive identification of *C. tropicalis* and other organisms using BCID and BCID2 panels. This may be related to yeast DNA in the culture media. *C. tropicalis* has been detected from 18 additional blood cultures up to March 2024 from multiple batches of blood culture bottles and multiple BCID and BCID2 panels.

**Risk management:**

Laboratory practices were reviewed to address the possibility of sample contamination. Of note, *C. tropicalis* was not cultured from blood in the laboratory in the year prior August 2023. All scientific and medical staff in the laboratory were alerted to the issue. Use of the BCID and BCID2 panels was continued because of the clinical value of rapid on-site identification of key pathogens such as *S. aureus* and differentiation from CoNS. In each case of *C. tropicalis* identification, the Gram stain and clinical features were reviewed. No patients received inappropriate antifungal treatment.

**Lessons for trainees:**

(i) The combination of two technologies (blood culture and multiplex NAAT) can generate unanticipated challenges. (ii) Use of NAATs in samples with a low pre-test probability is associated with the risk of low positive predictive value. (iii) Potential risk can be mitigated by correlation of results of molecular testing with Gram stain and assessment of clinical features.

**Conclusions:**

Learning for microbiology trainees relates to the need for caution in interpretation of new technologies and the importance of pre-test probability in interpreting a positive result in any assay.

